# Therapeutic effects of percutaneous coronary intervention on acute myocardial infarction complicated with multiple organ dysfunction syndrome

**DOI:** 10.12669/pjms.35.6.1162

**Published:** 2019

**Authors:** Dajun Qian, Daqiong Zhou, Huan Liu, Di Xu

**Affiliations:** 1 Dajun Qian, Department of Cardiology, Wuxi People’s Hospital Affiliated to Nanjing Medical University, Qingyang Road No. 299, Wuxi 214023, Jiangsu Province, China; 2 Daqiong Zhou, Department of Cardiology, Wuxi People’s Hospital Affiliated to Nanjing Medical University, Qingyang Road No. 299, Wuxi 214023, Jiangsu Province, China; 3 Huan Liu, Department of Cardiology, Wuxi People’s Hospital Affiliated to Nanjing Medical University, Qingyang Road No. 299, Wuxi 214023, Jiangsu Province, China; 4 Di Xu, Jiangsu Province Hospital, Nanjing 210029, Jiangsu Province, China

**Keywords:** Multiple organ failure, Myocardial infarction, percutaneous coronary intervention, Treatment efficacy

## Abstract

**Objective:**

To evaluate the clinical efficacy of percutaneous coronary intervention (PCI) on patients with acute myocardial infarction (AMI) complicated with multiple organ dysfunction syndrome (MODS).

**Methods:**

A total of 216 patients with AMI complicated with MODS enrolled from January 2016 to March 2018 were divided into a PCI group (n=98) and a drug treatment group (n=118). The baseline clinical data, the incidence of each dysfunction organ, the number of dysfunctional organs and the mortality were compared between the two groups.

**Results:**

The number of patients with ST-segment elevation AMI in the PCI group was higher than in the drug treatment group, and the rate of patients with non-ST-segment elevation AMI was lower than in the drug treatment group (P<0.05). The use of temporary pacemakers and IABP was similar between the two groups (P>0.05). The recanalization rate in PCI group was much higher than that in the drug treatment group (P<0.05). The two groups had similar incidence of organ dysfunction in the heart, lungs, kidneys, stomach and intestine, etc. and the PCI group had lower organ dysfunction incidence in the liver, brain and hematological system than the drug treatment group (P<0.05). The dysfunction incidence rate of two organs was higher in PCI group than in drug treatment group (P<0.05), the dysfunction incidence rate of 3 organs was similar between the two groups, and the dysfunction incidence rate of three organs or more was significantly lower in PCI group than in drug treatment group (P<0.05).

**Conclusion:**

Despite the high risk and high mortality of patients with AMI plus MODS, clinical improvement can still be achieved when effective PCI is performed.

## INTRODUCTION

AMI refers to severe myocardial ischemia and necrosis due to the acute stenosis or occlusion of the coronary arteries which can lead to continuously reduced or even terminated blood supply. In terms of the pathophysiological mechanism, AMI is mainly the result of vulnerable plaque rupture and thrombus formation and severe acute stenosis or complete occlusion of the coronary arteries induced by certain mechanical factors (such as hypertension, coronary spasm, etc.) in addition to coronary atherosclerosis.^1^ AMI is common among the mid-aged and elderly population with more men suffering than women. It can also be found in young patients. This disease is a dangerous type of coronary heart disease featuring rapid onset, dangerous complications high mortality, and poor prognosis.^2^ Patients with acute myocardial infarction (AMI) will suffer from acute myocardial ischemia and hypoxia as well as decreased cardiac output, leading to hypoperfusion in various organs and multiple organ dysfunction syndrome (MODS) in a very short period of time. If not promptly corrected, this disease can develop into irreversible multiple organ failure (MOF) with significantly increased mortality.^3^ AMI complicated with MODS used to be deemed as a relative contraindication of percutaneous coronary intervention (PCI).

However, the problem of hypoperfusion of the organs cannot be solved unless the infarct-related blood vessels are canalized promptly to restore the myocardial contractility.^4^ With this background the efficacy of PCI in patients with AMI complicated with MODS enrolled in our hospital from January 2016 to March 2018 was evaluated.

## METHODS

This study had been approved by the ethics committee of our hospital, (Acceptance letter dated July 4, 2018) and written consent was obtained from all patients. Retrospective analysis was done for a total of 216 subjects with AMI (including ST-segment elevation AMI and non-ST-segment elevation AMI) complicated with MODS. MODS was successively found in all patients after the onset of AMI. The patients were divided into an emergency PCI surgical treatment group and a drug treatment group based on their accessibility to PCI. Ninety-eight patients in the PCI group were subjected to emergency PCI, and 118 patients were in the drug treatment group and were treated with routine drug therapy but not PCI.

The drug treatment group received conventional oxygen therapy, sedation, vital sign maintenance, thrombolysis, organ protection and other supporting therapies. The PCI group received coronary intervention in addition to drug treatment. The patients took aspirin (300 mg,) and clopidogrel (300-600 mg,) routinely before PCI**.** The infarct-related arteries (IRAs) were determined with emergency conventional coronary angiography. In any case where the IRA lesion residual stenosis was greater than or equal to 50% and the thrombolysis in myocardial infarction (TIMI) blood flow was less than or equal to Level-2, the lesion stenosis and reference vessel diameter should be visually observed according to the diameter of the guiding catheter, and emergency PCI should be performed with conventional methods promptly. PCI was considered successful only when the IRA residual stenosis was less than or equal to 20% and the TIMI blood flow reached Level-3. The patients were subject to conventional subcutaneous low-molecular-weight heparin injection (40-60 mg/12 h, 3-7 d), and oral administration of aspirin (300 mg/d) and clopidogrel (75 mg/d) after the intervention. Patients in the two groups with bradyarrhythmia were subject to temporary pacing therapy, and those with severe hemodynamic instability were subject to intra-aortic balloon pumping (IABP).

### Statistical Analysis

The categorical data were expressed as mean ± standard deviation (x ± s), and the numerical data were expressed as percentages. The categorical data were analyzed by the non-paired t test, and the numerical data were analyzed by the Chi-square test. All data were statistically analyzed by SPSS 15.0 software.

## RESULTS

### Baseline clinical characteristics

The PCI group had 52 male subjects (53.06%), 66 elderly subjects (years old) (67.35%), and 55 Killip class IV subjects (56.12%); and the drug treatment group had 64 male subjects (54.34%), 82 elderly subjects (69.49%), and 61 Killip class IV subjects (51.69%). There was no significant difference in the frequency rates of hypertension, diabetes, cerebrovascular diseases, and myocardial infarction between the two groups (P>0.05). The rate of patients with ST-segment elevation AMI in the PCI group was higher than in the drug treatment group, and the rate of patients with non-ST-segment elevation AMI was lower than in the drug treatment group (P<0.05). The use of temporary pacemakers and IABP was similar between the two groups (P>0.05). The recanalization rate in PCI group was much higher than that in the drug treatment group (P<0.05) (Table-I).

**Table I:** General clinical information of the two groups [case (%)].

Item	PCI (n=98)	Drug treatment (n=118)	P-value
Male	52 (53.06%)	64 (54.34%)	0.552
≥60 years old	66 (67.35%)	82 (69.49%)	0.464
Hypertension	71 (72.45%)	87 (73.73%)	0.582
Diabetes	51 (52.04%)	62 (52.54%)	0.641
Cerebrovascular disease	39 (39.79%)	48 (40.68%)	0.586
ST-segment elevation AMI	88 (89.80%)	61 (51.69%)	0.006
Non-ST-segment elevation AMI	10 (10.20%)	57 (48.31%)	0.004
Killip class IV	55 (56.12%)	61 (51.69%)	0.278
***Infarction position***			
Anterior	23 (23.47%)	31 (26.27%)	0.367
Extensive anterior	38 (38.78%)	42 (35.59%)	0.386
Anterior + inferior	12 (12.24%)	14 (11.86%)	0.408
Right ventricle + inferior	16 (16.33%)	21 (17.80%)	0.417
Inferior + right ventricle + posterior	6 (6.12%)	6 (5.08%)	0.399
Anterior + inferior + right ventricle	3 (3.06%)	4 (3.39%)	0.526
Temporary pacing	11 (11.22%)	9 (7.63%)	0.192
IABP	16 (16.33%)	15 (12.71%)	0.188
Vascular recanalization rate	92 (93.88%)	65 (55.08%)	0.008

PCI: Percutaneous coronary intervention, IABP: Intra-aortic balloon pumping, AMI: Acute myocardial infarction.

### Incidences of organ dysfunction

The emergency PCI group had 98 patients with heart failure (100%), 93 with respiratory failure (94.90%), 22 with renal dysfunction (22.45%), and 12 with gastrointestinal dysfunction (12.24%). These data showed no significant difference between the two groups (P>0.05). Meanwhile, the PCI group had significantly lower incidence rates of liver, brain and blood system dysfunctions than the drug treatment group (P<0.05). The elderly patients in the PCI group had lower incidence of brain and blood system dysfunctions than the drug treatment group (P<0.05) and higher incidence rate of gastrointestinal dysfunction than the drug treatment group (P<0.05) (Table-II).

**Table II:** Organ dysfunction incidences of the two groups [case (%)].

Organ	PCI (n=98)	Drug treatment (n=118)	P value
Heart	98 (100%)	118 (100%)	
Lung	93 (94.90%)	106 (89.83%)	0.144
Kidney	22 (22.45%)	21 (17.80%)	0.117
Liver	14 (14.29%)	31 (26.27%)	0.009
Intestines and stomach	12 (12.24%)	11 (9.32%)	0.207
Brain	8 (8.16%)	22 (18.64%)	0.024
Hematological system	2 (2.04%)	11 (9.32%)	0.037

PCI: Percutaneous coronary intervention, IABP: Intra-aortic balloon pumping, AMI: Acute myocardial infarction.

### Number and prognosis of dysfunctional organs

The dysfunction incidence rate of 2 organs was higher in PCI group than in drug treatment group (P<0.05), the dysfunction incidence rate of three organs was similar between the two groups, and the dysfunction incidence of three organs or more was significantly lower in PCI group than in drug treatment group (P<0.05). The mortality rates in the both groups increased with the increase of the numbers of organs with dysfunction. Both the mortality rate with dysfunction incidence of two, three or four organs and the overall mortality rate were significantly lower in the PCI group than in the drug treatment group (P<0.05). The mortality rates were very high in both groups when there were dysfunction incidences of five organs, the mortality rate in the PCI group being 66.67% and that in the drug treatment group being 75.00%. This mortality was lower in the PCI group than in the drug treatment group, but the difference was not statistically significant (P>0.05) (Table-III). Among elderly patients, the dysfunction incidence rate of two organs was higher in PCI group than in drug treatment group (P<0.05), the dysfunction incidence rate of three organs was similar between the two groups (P>0.05), and the dysfunction incidence rate of three organs or more was lower in PCI group than in drug treatment group (P<0.05). The mortality rate of elderly patients was significantly lower in the PCI group than in the drug treatment group (P<0.05).

**Table III:** Number and mortality rates of dysfunctional organs of the two groups [case (%)].

MODS Organ No.	Case No.			Death No.			Mortality rate (%)		

	PCI	Drug treatment	P-value	PCI	Drug treatment	P-value	PCI	Drug treatment	P-value
2	66 (67.35%)	63 (53.39%)	0.006	2 (25.00%)	8 (22.22%)	0.463	9 (13.64%)	24 (38.10%)	0.017
3	24 (24.49%)	25 (21.19%)	0.316	3 (37.50%)	14 (38.89%)	0.547	4 (16.67%)	11 (44.00%)	0.012
4	5 (5.10%)	18 (15.25%)	0.008	2 (25.00%)	10 (27.78%)	0.519	2 (40.00%)	15 (83.33%)	0.007
≥5	3 (3.06%)	12 (10.17%)	0.008	1 (12.50%)	4 (11.11%)	0.508	2 (66.67%)	9 (75.00%)	0.175

PCI: Percutaneous coronary intervention, IABP: Intra-aortic balloon pumping, AMI: Acute myocardial infarction.

## DISCUSSION

AMI is a common, acute and severe disease among cardiovascular diseases. Myocardial necrosis and decreased myocardial contractility lead to congestive heart failure and cardiogenic shock, leading to drop in blood pressure, decreased urine output, and microcirculatory perfusion disorders.^5^,^6^ Meanwhile, large amounts of catecholamines and vasopressin are released to worsen the myocardial ischemia and aggravate the ischemia and hypoxia of other organs. The cells damaged by hypoxia will have enzymatic changes, release of free oxygen radicals, generation of platelet-activating factors, and activation of complements, further damaging the cellular tissues and causing a chain of pathological responses as well as MODS.^7^,^8^ MODS is a severe life-threatening clinical syndrome. If not timely treated, it will develop into MOF with irreversible organ necrosis as well as significantly increased mortality.^9^ According to literature, the top four underlying diseases for MOF mortality are cardiovascular diseases, cancer, lung diseases and brain disorders, in which cardiovascular diseases are the most important cause of death.^10^ The intracellular hypoxia is widely recognized as the ultimate path of MOF.^11^

MODS complicating AMI often occurs successively in the heart, lungs, kidneys, liver, stomach and intestines, brain, blood system and others.^12^ The data show that when the baseline clinical characteristics were similar, the incidence of dysfunctions in the heart, lungs, kidneys and gastrointestinal organs were similar between the two groups; and the incidence of dysfunctions in liver, brain and blood system were significantly lower in the PCI group than in the drug treatment group. This indicates that heart failure, pulmonary edema and renal dysfunction occurred successively shortly after AMI. After the infarct-related blood vessels were recanalized with PCI, some of the ischemic myocardium got effective perfusion with improved cardiac function and increased ejection fraction as well as significantly increased blood flow volume in various organs. This reversed the continuous development of MODS and reduced the organ failures in the liver, brain and blood system.^13^,^14^ However, compared with the PCI group, the drug treatment group showed lower vascular recanalization rate and unrestored myocardial blood supply. The organs of patients in the drug treatment group were in low-perfusion state and the MODS could continue its development and lead to more damage to the organs. Among elderly patients, the dysfunction incidence of brain and blood system dysfunctions were lower in the PCI group than in the drug treatment group, and the incidence of gastrointestinal dysfunction was higher in the PCI group than in the drug treatment group. This suggests that the elderly patients will suffer from severe gastrointestinal mucosal injuries with oral administration of aspirin and clopidogrel during the emergency PCI. The gastrointestinal mucosal injuries can easily induce gastrointestinal mucosal erosion and hemorrhage, and gastric mucosal protective agents such as omeprazole can be used to reduce the incidence of gastrointestinal hemorrhage. Hypertension and cerebral arteriosclerosis are common among elderly patients. Therefore, the elderly patients were very prone to stroke in the drug thrombolysis with incidence significantly higher than the PCI group. MODS was aggravated by the incidence of stroke.^15^ The PCI group were treated with positive revascularization therapy and achieved increased vascular recanalization rate and restoration of myocardial blood supply. The development of MODS was reversed since the ischemia of more organs was shortened and the damage to organs was reduced.^16^

The key to treatment of patients with AMI complicated with MODS, especially elderly patients, is the full awareness of the risk factors and the early prevention of potential MODS.^13^ The data show that heart failure and respiratory failure occurring after AMI were the most common in the MODS, which is consistent with the literature. Their onsets were ferocious, suggesting that the heart is the key organ that should be treated in the early stage. One viable treatment method is the emergency PCI in combination with the conventional drug treatment. The history of gastrointestinal diseases especially the history of hemorrhage must be inquired in detail before the emergency PCI. IABP support should be used during intervention to improve myocardial perfusion and enhance the output capacity of the heart. Gastrointestinal mucosal protective agents should be administered in addition to the conventional drug treatment after the surgery. The elderly patients with decreased body resistance should be prevented from infection and provided with good care on diet, bowel function and chest physiotherapy**,** etc.^17^ The elderly patients account for more than 60% of the subjects in this study. The reserve function of vital organs decreases as the patient gets older. The elderly may also be affected by hypertension, diabetes, cerebrovascular diseases, systemic atherosclerosis and other diseases and can maintain some normal physiological functions when there is no extra pressure. When brought under some stress or extra pressure, the elderly patient would quickly enter a state of decompensation and be very prone to single organ failure and may further develop MOF.^18^ MODS used to be deemed as a relative contraindication of PCI. However, the infarcted myocardia cannot get perfusion, the myocardial contractile function cannot be restored, and the neighboring organs cannot get effective perfusion unless the infarct-related blood vessels are canalized.^19^ Some blood vessels can be canalized by drug thrombolysis whose canalization rate is however significantly lower than that of PCI.^20^

## CONCLUSION

Patients with AMI plus MODS have high risk and mortality, but effective PCI still exerts satisfactory therapeutic effects. Further studies based on larger sample sizes are in needed to optimize the therapy using PCI.

### Authors’ contributions:

**DZ, HL & DX** performed this study, analyzed clinical data and prepared this manuscript.

**DQ & DZ** designed this study and significantly revised this manuscript and they contributed equally to this study equally and are responsible for integrity of research.
